# miRNA in the Diagnosis and Treatment of Critical Limb Ischemia

**DOI:** 10.3390/biomedicines12092026

**Published:** 2024-09-04

**Authors:** Alexandra Ioana Popescu, Andreea Luciana Rață, Daliborca Vlad, Cristian Vlad, Roxana Popescu, Ramona Roxana Onofrei, Marialuisa Morelli, Stelian Pantea, Sorin Barac

**Affiliations:** 1 Pharmacology Department, “Victor Babes” University of Medicine and Pharmacy, 300041 Timisoara, Romania; alexandra.popescu@umft.ro (A.I.P.); vlad.daliborca@umft.ro (D.V.); vlad.cristian@umft.ro (C.V.); 2 Surgical Emergencies Department, “Victor Babes” University of Medicine and Pharmacy, 300041 Timisoara, Romania; pantea.stelian@umft.ro; 3 Cell and Molecular Biology Department, “Victor Babes” University of Medicine and Pharmacy, 300041 Timisoara, Romania; popescu.roxana@umft.ro; 4 Department of Rehabilitation, Physical Medicine and Rheumatology, Research Center for Assessment of Human Motion, Functionality and Disability, ”Victor Babes” University of Medicine and Pharmacy, 300041 Timisoara, Romania; onofrei.roxana@umft.ro; 5 Vascular Surgery Department, “Victor Babes” University of Medicine and Pharmacy, 300041 Timisoara, Romania; isamari1991@gmail.com (M.M.); sorinbarac@gmail.com (S.B.)

**Keywords:** chronic threatening limb ischemia, microRNA, atherosclerosis

## Abstract

Chronic threatening limb ischemia of the inferior limbs (CLTI) is the final stage of peripheral arterial disease (PAD) and is one of the most feared atherosclerotic manifestations because if left untreated, in time, it can lead to amputation. Although there are currently numerous treatment techniques, both open and endovascular, it is a pathology that has no underlying treatment. Therefore, current studies are very much focused on new therapeutic possibilities that can be applied in the early stages of the atherosclerotic process. In numerous studies in the literature, miRNAs have been identified as important markers of atherosclerosis. The present study aims to identify the expression of three miRNAs—*miR-199a, miR-20a*, *and miR-30c*—in patients with chronic limb-threatening ischemia in the pre- and post-revascularization periods. The aim of the study is to identify whether these three markers play a role in critical ischemia and whether they have the potential for future use in new treatments of this pathology.

## 1. Introduction

Chronic lower limb-threatening ischemia (CLTI) is considered the most serious form of peripheral arterial disease (PAD) and is associated with a major risk of amputation, major cardiovascular events, and death [1]. Studies in the literature have reported a mortality rate of 20% in the first 6 months after diagnosis and 50% at 5 years. Clearly, this mortality rate is correlated with other atherosclerotic conditions, including coronary and cerebral pathology [2]. Following the 2019 European Society for Vascular Surgery guidelines for critical ischemia, a clear and concise definition was received, including a heterogeneous group of patients with varying degrees of ischemia that prevents lesion healing and who are at increased risk of amputation. Thus, the diagnosis of critical ischemia now requires the diagnosis of peripheral arterial disease in association with resting pain and tissue loss (ulceration or gangrene) [3].

There is currently a complex array of clinical and paraclinical methods for determining CLTI, but there is very little information about the biomarkers of CLTI [4].

miRNAs have been identified as markers of atherosclerotic disease. miRNAs are short, single-stranded RNA fragments of approximately 22 nucleotides that can modulate gene expression post-transcriptionally [5]. A recent series of studies demonstrated their utility as biomarkers of CLTI and raised the question about their potential in the treatment of disease [6].

Several miRNAs are being explored as potential markers for predicting and evaluating peripheral arterial disease, but the current data are inconclusive, and more research is needed. There is also a lack of studies on the relationship between circulating miRNAs and clinical parameters in critical limb ischemia. More than 600 miRNAs have now been identified and are thought to regulate more than 50% of human protein-coding genes. miRNAs are, in fact, as functionally important as a transcription factor.

Endothelial cells are one of the most important factors in the health of the vascular tree. There are several miRNAs identified that play an important role in angiogenesis: miR21, the overexpression of which reduces endothelial cell proliferation, reduced actin organization in stress fibers (which may play a role in decreasing cell migration) [7] or miR-126 overexpression, which inhibits leukocyte adhesion to endothelial cells [8].

Other miRNAs have a role in smooth muscle cells: miR-1 overexpression inhibits proliferation, and miR-21 is a proproliferative and antiapoptotic regulator [9].

The aim of this study was to examine the expression of three miRNAs in CLTI patients, both before and after revascularization procedures. The goal is to enhance our understanding of the role of miRNAs in the clinical management of PAD.

## 2. Materials and Methods

This study was conducted in the Vascular Surgery Department of the “Pius Brînzeu” County Clinical Hospital, Timișoara, Romania. We enrolled 21 patients diagnosed with critical limb-threatening ischemia (CLTI). We collected data 24 h prior to and after the revascularization procedure, with either an open or endovascular one.

The inclusion criteria were as follows: patients above 18 years of age who agreed to participate in the study and confirmed with CLTI according to the current ESVS guidelines. (Ischemic rest pain is usually described as affecting the forefoot and usually aggravated by the decubitus position; for the diagnosis, the pain should be present for more than 2 weeks and associated with one or more abnormal parameters: ankle-brachial index < 0.4, ankle pressure < 50 mmHg in absolute value, hallux pressure under 30 mmHg for the absolute value, a transcutaneous oxygen pressure under 30 mmHg and a flat or weakly pulsating wave curve (equivalent to the third degree of ischemia in the WIfI—Wound, Ischemia, foot Infection classification).The exclusion criteria were patients under 18 years old or who did not give their consent for participation, patients with previous revascularization (no more than 3 months) on the index leg, patients on steroid medication, pregnancy, chronic kidney disease, active neoplasia, chronic liver disease Child–Pugh B minimum, thrombophilia, and other autoimmune diseases that could enhance the microRNA expression.

We also documented all patients’ comorbidities, such as coronary disease, hypertension, diabetes mellitus, smoking habits, and medication used pre-admission.

All participants in the study read and signed an informed consent. The data were collected under GDPR (General Data Protection Regulation) laws. The study obtained an agreement from the Hospital Ethics Committee, under the EU GCP Directives, the International Conference of Harmonization of Technical Requirements for Registration of Pharmaceuticals for Human Use (ICH), and the Declaration of Helsinki.

### miRNA Collection

Blood samples were meticulously collected in standard vacutainer tubes, each containing EDTA, which acted as an effective anti-coagulant to prevent clotting. We investigated three miRNAs: miR-199a, miR-20a, and miR-30c.

The procedure for miRNA isolation from the collected blood samples was carried out using the MagMax mirVana™ Total RNA Isolation kit, specifically provided by Applied Biosystems and Thermo Fisher Scientific, Baltics UAB (catalog number A27828) (Hillsboro, OR, USA). This kit is known for its efficiency in isolating high-quality total RNA.

Following the isolation process, the quantity of RNA was measured using the Qubit 4 fluorometer, which provides precise quantification. The measurement was performed with the aid of the Qubit™, RNA assay kit, also from Invitrogen by Thermo Fisher Scientific, (Hillsboro, OR, USA) (catalog number Q32880). This kit is well-regarded for its sensitivity and accuracy in RNA quantification. A specific concentration of RNA, measured at 8 ηg/µL, was then converted into complementary DNA (cDNA). This conversion was achieved through the use of the TaqMan™, MicroRNA Reverse Transcription kit, again sourced from Applied Biosystems by Thermo Fisher Scientific, Baltics (catalog number 4366597). This particular kit is designed for the efficient reverse transcription of miRNA into cDNA.

For the purpose of real-time polymerase chain reaction (RT-PCR) amplification, the TaqMan™ Universal Master Mix II, Baltics UAB with UNG was utilized, which was provided by the same manufacturer (catalog number 4440042). This master mix is known for its reliability in various PCR applications.

Additionally, the TaqMan™ miRNA Assay, including specific assays for has-miR-20a, has-miR-30c, and has-miR-199a, was employed to facilitate the amplification of the miRNA of interest. These assays are recognized for their specificity and sensitivity in detecting miRNA.

The cDNA template, prepared from the RNA, was then used in conjunction with the QuantStudio5 Real-Time PCR system, Singapore provided by Applied Biosystems (Waltham, MA, USA) by Thermo Fisher Scientific, to carry out the amplification process. This system is known for its high precision and reproducibility in quantitative PCR.

Each sample underwent testing in duplicate to ensure the accuracy and reliability of the results. This duplication helps in mitigating any potential variability and ensures the robustness of the data.

The relative expression levels of miRNA were determined by calculating the cycle threshold (Ct) values and the delta Ct (∆Ct) values. The ∆Ct value is defined as the difference between the Ct value of the housekeeping gene U6 miRNA and the Ct value of the target miRNAs. A low delta Ct value indicates high miRNA expression, suggesting that a small number of fractional cycles were required to reach the amplification threshold for the target miRNAs. Conversely, a high delta Ct value signifies the low expression of target miRNAs, reflecting a greater number of cycles needed to reach the threshold. 

For the analysis of the data and statistical validation of the results, GraphPad Prism version 10.2.2 was used, which is widely recognized for its comprehensive statistical analysis capabilities. Paired *t*-tests were performed between pre- and post-treatment differences based on lower limb viability. Differences were considered statistically significant at *p* < 0.05. We also used a ROC (receiver-operating character) curve to determine the predictive performance of miRNAs.

## 3. Results

Twenty-one patients aged 43–81 years old (mean age 62.50 ± 7.88) were included in the study from the Department of Vascular Surgery. There were five females (23.8%) and sixteen males (76.2%). Patients’ demographics, comorbidities, risk factors, and other relevant data are presented in Table 1. One can observe that all the comorbidities are highly specific for patients with chronic limb-threatening ischemia, with the majority of patients having coronary disease, hypertension, and diabetes mellitus.

After the evaluation of the three microRNAs, descriptive statistics on the expression levels of microRNA miRNA30c, miRNA199a, and miRNA20a before and after surgery emerged.

miRNA30c and miRNA20a demonstrate significant changes in expression post-surgery, with miRNA30c showing a reduction and miRNA20a exhibiting a notable shift closer to zero, both indicating their potential as diagnostic markers. miRNA199a, while showing an increase in expression post-surgery, did not achieve the same level of statistical significance (Table 2).

The ΔCt value is a measure of the expression level of a gene, with lower ΔCt values indicating higher expression. These graphs are useful for visualizing the changes in gene expression levels across different conditions or time points.

**Figure 1:**
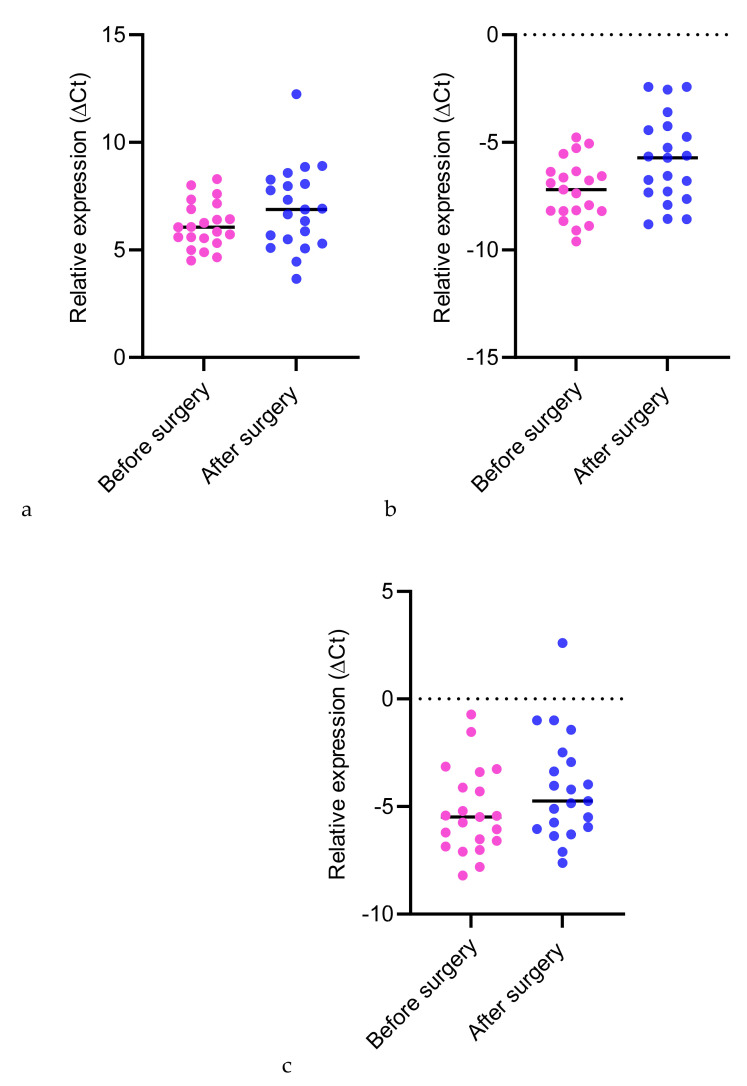
miRNA199a (**a**), miRNA20a (**b**) and miRNA30c (**c**) relative expression graph.

Figure 1 shows the miRNA199a expression before and after the revascularization procedure. The mean ΔCt value before surgery is approximately 6.152 with a standard deviation of 1.076, while after surgery, the mean ΔCt value increases to 6.924 with a standard deviation of 1.937. This suggests a slight increase in miR-199a expression after surgery, though the change is not statistically significant—*p* = 0.0751.

For miRNA20a, the mean ΔCt value before surgery is −7.221 with a standard deviation of 1.38. After surgery, the mean ΔCt value increases to −5.848 with a standard deviation of 2.038. The change in miR-20a expression is statistically significant, with a *p*-value of 0.0078, indicating a significant increase in expression post-surgery.

For miRNA30c, the mean ΔCt value before surgery is −5.243 with a standard deviation of 1.978, and after surgery, the mean ΔCt value increases to −4.149 with a standard deviation of 2.462. This change is statistically significant (*p* = 0.0319), indicating an increase in miR-30c expression after surgery. 

Summarizing this, miRNA30c and miRNA20a demonstrate significant changes in expression post-surgery, with miRNA30c showing a reduction and miRNA20a exhibiting a notable shift closer to zero, both indicating their potential as diagnostic markers. miRNA199a, while showing an increase in expression post-surgery, did not achieve the same level of statistical significance. The ROC curves further highlight miRNA30c as the superior diagnostic tool among the three, followed by miRNA20a and miRNA199a. These findings underscore the importance of miRNA expression changes in the context of surgical interventions and their potential utility in diagnostic applications.

**Figure 2:**
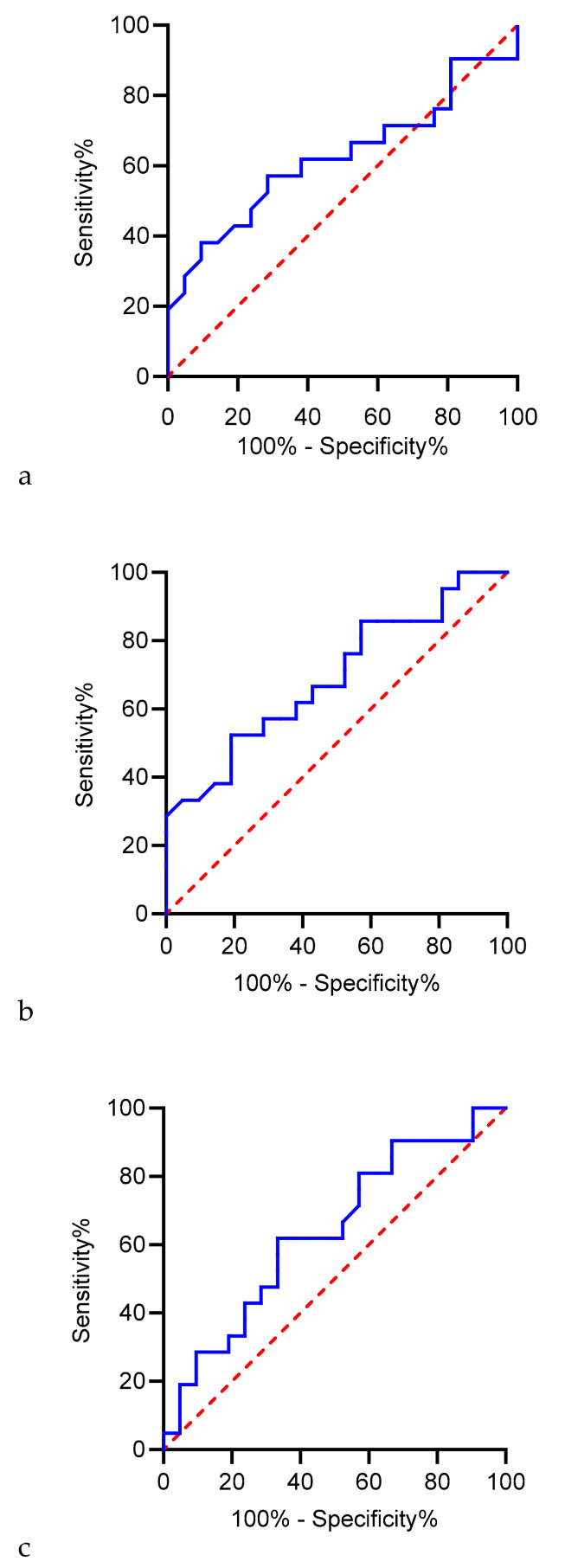
miRNA199a (**a**), miRNA20a (**b**), and miRNA30c (**c**) ROC curves.

The ROC curve (Figure 2) for the different miRNAs in this study demonstrates the diagnostic performance of this biomarker with specific numerical data and reveals significant insights into the diagnostic efficacy of this particular biomarker.

The ROC curve for miRNA199a shows that it performs only slightly better than random guessing, indicating limited diagnostic potential. Its performance, closely resembling that of a random classifier, indicates that miRNA199a is not a robust biomarker for the conditions under investigation; thus, it is crucial to identify biomarkers with a higher AUC and a more significant ability to differentiate between positive and negative cases, ensuring more reliable and effective diagnostic outcomes.

The area under the curve (AUC) appears to be significantly greater than 0.5, suggesting that miRNA 20a has good discriminatory power for the classification task at hand. The curve’s overall proximity to the top-left corner, particularly in its initial steep ascent, indicates robust performance, demonstrating that the classifier achieves high sensitivity with relatively low false-positive rates.

The area under the curve (AUC) appears to be greater than 0.5, indicating that miRNA 30c has some distinguishing power. However, it does not appear to be as strong as it could be (the curve lies near the diagonal baseline), which implies moderate to marginal improvement over random guessing. The curve does not exhibit a rapid ascent toward the top-left corner, which could indicate higher sensitivity at lower false-positive rates.

## 4. Discussion

The atherosclerotic process is a pathology that develops over many years of life and may be asymptomatic for a long time, but when progression occurs, it becomes one of the major factors in cardiovascular mortality. Atherosclerosis manifests itself in several forms, causing stroke, acute myocardial infarction, and peripheral arterial disease with all its manifestations, both in the limbs and in other peripheral vascular territories. Most often, in the case of the lower limbs, chronic limb-threatening ischemia is caused by the instability of the atheromatous plaque, favoring its rupture, thrombosis formation at its level, and critical ischemia.

Modern research has recently been very much oriented towards developing strategies to identify vulnerable atherosclerotic plaques.

The miR-199 family consists of three members—miR-199a1, miR-199a2, and miR-199b—and functional studies in recent years have shown that they play an important role in maintaining normal homeostasis and regulating disease pathogenesis [10].

MiRNA199a, which has multiple roles in vascular biology, plays a significant role in the context of PAD pathophysiology due to potential therapeutic implications. The effects that can impact the regulation of PAD progression and treatment are as follows: the migration and proliferation of VSMC (vascular smooth muscle cells), which play a crucial role in the formation of atherosclerosis, are regulated by miRNA199a via a control. Thus, when slowing the development of new plaque and stabilizing present plaques, the course of PAD is slowed down. Atherosclerosis, one of the main causes of PAD, is diminished by VSMC [11]. Additionally, miRNA199a aids in the preservation of activity in endothelial cells, which are essential for vascular health. PAD can worsen due to the increased vascular inflammation and plaque formation caused by dysfunctional endothelial cells. miRNA199a promotes healthy endothelium function, and in that way, the risk of vascular problems related to PAD decreases [11]. By regulating the expression of pro-inflammatory cytokines and molecules, miRNA199a has anti-inflammatory effects. One of the main causes of PAD and atherosclerosis is inflammation. Thus, miRNA199a’s anti-inflammatory qualities may contribute to reducing the inflammatory load in arteries, leading to better results for PAD patients [12].

The mean ΔCt value for miRNA199a post-surgery increased to 6.924, suggesting an increase in the expression levels of miRNA199a after surgery. The standard deviation increased to 1.937, indicating more variability in post-surgery miRNA199a expression levels. The standard error of the mean rose to 0.4228, reflecting greater variability in the sample’s representation of the population mean. The median value after surgery was 6.88, indicating a central tendency towards higher ΔCt values. However, the *p*-value for miRNA199a was 0.0751, which is above the 0.05 threshold, indicating that the changes in miRNA199a expression are not statistically significant. Liu et al. demonstrated the down-regulation of miRNA-199a-5p in smooth muscle cells within the arterial wall in patients with restenosis after vascular procedures. Also, in vivo, the delivery of this microRNA via a lentivirus led to neointimal proliferation after angioplasty procedures, making this miRNA a therapeutic target in these patients [13].

A study led by Qiao et al. demonstrated that miR-199a is a pro-atherogenic miRNA, and its overexpression is positively correlated with the atherosclerotic process, and in particular with endothelial cell damage. In fact, miR-199a induces phenotypic abnormalities in endothelial cells, such as proliferation, migration, and tube formation, by mediating pro-inflammatory axes [14].

MiR-17, miR-18, miR-19a, miR-19b, miR-20a, and miR-92a are the members of the miR-17–92 family [15,16]. The miR-17–92 cluster, which—as mentioned above—includes miRNA20a, is well-known for its role in carcinogenic processes [17]. This cluster is generally considered to be a well-researched miRNA cluster. miRNA20a binds on its target genes more specifically compared to the untranslated regions of the 3′ site (3′ UTR) of the target mRNAs, which are degraded or undergo translational suppression [18].

After surgery, the mean ΔCt value for miRNA20a decreased to −5.848, indicating a reduction in the expression levels of miRNA20a post-surgery. The standard deviation increased to 2.038, suggesting greater variability in miRNA20a expression levels among patients after surgery. The standard error of the mean rose to 0.4448, reflecting a broader range in the sample’s representation of the population mean. The median value post-surgery was −5.72, which, although higher than the pre-surgery median, still indicates a negative ΔCt value, reflecting the continued but reduced expression of miRNA20a. The *p*-value associated with the change in inmiRNA20a expression was 0.0078, which is less than the threshold of 0.05, indicating that the observed changes in miRNA20a expression before and after surgery are statistically significant.

Within the miR-17/92 cluster, miR-92a and miR-17 have been particularly studied in cardiovascular pathology, with the overexpression of miR-92a in ischemic cells leading to the inhibition of angiogenesis. Another study on ischemic coronary artery disease demonstrated increased levels of both miR-92a and miR-17 in endothelial cells compared to healthy subjects. Although the expression of miR-20a has not been studied much in the literature, our study places it in a new light, given the statistical significance it demonstrated. Moreover, it can be evaluated as a marker of restenosis post-revascularization procedures, making it an important marker in chronic limb-threatening ischemia [19].

The miR-30 is a miRNA family that consists of six miRNA molecules, which are mature: miR-30a, miR-30b, miR-30c-1, miR-30c-2, miR-30d, and miR-30e. They form a crucial subset of miRNAs with important roles in cardiovascular biology. Encoded by six genes, they are spread over three different regions of chromosomes: 1p34.2 (site of miR-30c and miR-30e), 8q24.22 (site of miR-30b and miR-30d), and finally 6q13 (site of miR-30a). Although they have the same 5′ sequence, they differ at the end of the 3′ sequence, allowing them to carry out a variety of biological tasks [20]. Their involvement in PAD and their importance in preserving vascular health by their regulation of vascular smooth muscle cell behavior and endothelial function tackles the primary underlying cause of PAD, atherosclerosis. They also assist in lowering inflammation and oxidative stress in the vascular system in order to preserve the integrity and function of the arteries [21].

Apart from that, miRNA30c significantly impacts lipid metabolism. One of its main targets,¸ an essential enzyme involved in the assembly of lipoproteins and secretions, is the “microsomal triglyceride transfer protein” (MTTP). By inhibiting MTTP, which lowers plasma lipid levels, miRNA30c effectively lowers the secretion of triglycerides and very low-density lipoprotein (VLDL). Thus, miRNA30c protects against lipid abnormalities, which are fundamental to atherosclerosis and other CVDs [22,23].

After surgery, the mean ΔCt value for miRNA30c increased to −4.149, indicating a reduction in the expression levels of miR30c post-surgery. The standard deviation also increased to 2.462, suggesting greater variability in miRNA30c expression levels among patients after surgery. The standard error of the mean rose to 0.5372, reflecting a broader range in the sample’s representation of the population mean. The median value post-surgery was −4.75, which, although higher than the pre-surgery median, still indicates a negative ΔCt value, reflecting the continued but reduced expression of miRNA30c. The *p*-value associated with a change in miRNA30c expression was 0.0319, which is less than the threshold of 0.05, indicating that the observed changes in miRNA30c expression before and after surgery are statistically significant. A study conducted by Zhang demonstrated that the expression of miR-30C in atherosclerotic vessels is much lower than in normal arteries, and this miRNA inhibits the differentiation of vascular smooth muscle cells from adventitial progenitor cells [24].

An important aspect to be mentioned and studied is the association of these miRNAs with cardiovascular risk factors leading to the development of peripheral arterial disease and its progression to critical ischemia.

There are numerous studies associating miRNA-199a expression with blood pressure. A study conducted by Lynch et al. demonstrated a statistically significant correlation in all 75 patients investigated [25]. Our study sought to determine whether these three miRNA markers play a role in critical lower limb ischemia. One of the limitations of this study is the small number of subjects included. However, we believe that this is an important starting point for assessing the expression of these markers in this pathology and is the basis for larger studies that will highlight miRNA expression in peripheral arterial disease and chronic limb-threatening ischemia in particular.

An important point to take into consideration is the fact that the expression changed after the revascularization procedure. Other studies showed that any changes in the blood flow (only through walking) induced changes in miRNA expression [26].

Ever since their identification, miRNAs have been regarded as having a key role in regulating vascular expression, both under normal and pathological conditions [27].

Their role in atherosclerosis, the leading cause of critical ischemia, comprises the fact that miRNAs are thought to participate either positively or negatively in almost all molecular processes involved in atherosclerosis and arterial remodeling, including endothelial dysfunction, monocyte activation, smooth muscle cell activation, and platelet activation [28,29].

Another important phenomenon to mention is the role of miRNAs in restenosis after revascularization procedures. It is important to note from our study that the miRNAs studied varied pre- and post-revascularization procedures. In fact, any revascularization procedure produces a mechanical vascular injury; first, there is an impairment of the vascular non-endothelium with the destruction of endothelial cells, a reendothelialization process, and the generation of new endothelium. This phenomenon is followed by an inflammatory response, with platelet activation, leukocyte attraction to the injury site, and the release of inflammatory factors and cytokines. This is followed very rapidly by the proliferation of smooth muscle cells and then tissue remodeling in which the smooth muscle cells enter into a hyperactive phase in which they are involved in depositing an extracellular matrix in the intima of the vessels [30].

Thus, basically, the protection of the vascular wall from treatment-induced injury actually leads to neointimal proliferation, vascular remodeling, and restenosis in the vessel.

In view of the above, studies at the level of miRNAs have demonstrated that they are responsible for a whole host of activities at the cellular level, noting here the proliferation of smooth muscle cells, the migration of leukocytes and platelets, and neointima formation.

If we take into account the two aspects mentioned in that miRNA expression changes only after walking exercises in patients with intermittent claudication, or after revascularization procedures, either open or interventional, we can strongly affirm that these markers are extremely important in peripheral arterial disease. Even if most of the current studies in this direction are small, with no control groups or validation cohorts, they still remain a solid basis for further investigations. Of course, uniform conditions, quantifiable and measurable outcomes, multicenter randomized trials, as well as large prospective studies are still needed.

## 5. Conclusions

The present study demonstrates the importance of microRNAs in chronic limb-threatening ischemia and their potential as biomarkers. Dynamics in their expression in different pre- and post-revascularization situations support the hypothesis that they may also have an important role in the treatment of this pathology when targeting their regulation.

The study of miRNA is a new concern in chronic limb-threatening ischemia. Therefore, different markers are still being identified, and it is necessary to further demonstrate in larger population-based studies which are the most relevant for diagnosis and which may have potential in treatment.

## Figures and Tables

**Table 1:** Demographics.

Item	Values
Age	67.9 ± 7.88
Gender (males)	85.72%
Coronary disease	85.71%
Hypertension	95.23%
Smoking	95.23%
Cardiac insufficiency	80.95%
Diabetes mellitus	61.90%
Pre-op medication	
Clopidogrel	42.85%
Asprin	90.47%
Rivaroxaban 2.5 bid	28.57%
Novel anti-coagulants	14.28%
Different associations	37.65%

**Table 2:** miRNAs value before and after surgery.

Descriptive Statistics	∆Ct		
	miR30c	miR199a	miR20a
Before surgery			
Mean	−5.243	6.152	−7.221
Std. Deviation	1.978	1.076	1.38
Std. Error of Mean	0.4315	0.2348	0.3012
Median	−5.48	6.06	−7.19
After surgery			
Mean	−4.149	6.924	−5.848
Std. Deviation	2.462	1.937	2.038
Std. Error of Mean	0.5372	0.4228	0.4448
Median	−4.75	6.88	−5.72
*p*-two tailed (Wilcoxon matched-pairs signed rank test)	0.0319	0.0751	0.0078

## Data Availability

The raw data supporting the conclusions of this article will be available upon request.
